# Healthcare workers and students in Bulgaria: Perspectives on Assisted Suicide

**DOI:** 10.1192/j.eurpsy.2025.1467

**Published:** 2025-08-26

**Authors:** G. Dogancali Yanchev, P. G. Chumpalova-Tumbeva, E. M. Dimitrova-Ilieva

**Affiliations:** 1 First Psychiatry Clinic, UMHAT Dr Georgi Stranski, Pleven EAD, Pleven, Bulgaria

## Abstract

**Introduction:**

This ongoing research investigates the attitudes of healthcare workers and students in Bulgaria toward assisted suicide, a practice that remains illegal and ethically debatable in the country.

**Objectives:**

The purpose of the research is to better understand how Bulgarian healthcare workers and students deal with assisted suicide’s ethical dilemmas and its potential impact on the country’s ability to provide end-of-life care in the future.

**Methods:**

Using mixed method approach including quantitative surveys, qualitative interviews, focused groups, this research seeks to capture wide range of opinions from healthcare professionals including medical doctors, nurses, laboratory assistants, midwifes, rehabilitators, medical students, nursing students, midwifing students etc.

**Results:**

Initial findings suggests that majority of the respondents of the focused group are opposed to the legalization of the assisted suicide due to ethical concerns including adherence of the Hippocratic Oath, potential misuse, and the influence of religious beliefs. The focus group does, however, include a small but notable percentage of individuals especially younger healthcare professionals and students who recognize, value, and appreciate the significance of patient autonomy and the elevation of suffering as essential components of compassionate care.

**Conclusions:**

This ongoing research into the views of healthcare workers and students in Bulgaria on assisted suicide uncovers a complex situation that is heavily influenced by ethical considerations and long-standing cultural and religious values. As the study progresses, it aims to dive deeper into how factors as age, duration of experience in medical field, specialty in medical field and social status contribute to these diverse views.

**Disclosure of Interest:**

None Declared

